# Classification of neuroendocrine neoplasms: lights and shadows

**DOI:** 10.1007/s11154-020-09612-2

**Published:** 2020-11-09

**Authors:** Stefano La Rosa, Silvia Uccella

**Affiliations:** 1 Institute of Pathology, University Hospital and University of Lausanne, Lausanne, Switzerland; 2 Institut Universitaire de Pathologie, CHUV, 25 rue du Bugnon, CH-1011 Lausanne, Switzerland; 3 Unit of Pathology, Department of Medicine and Surgery, University of Insubria, Varese, Italy

**Keywords:** Neuroendocrine neoplasm, Neuroendocrine tumor, Neuroendocrine carcinoma, Classification

## Abstract

Neuroendocrine neoplasms (NENs) are a heterogeneous group of neoplastic proliferations showing different morphological features, immunophenotype, molecular background, clinical presentation, and outcome. They can virtually originate in every organ of the human body and their classification is not uniform among different sites. Indeed, as they have historically been classified according to the organ in which they primarily arise, the different nomenclature that has resulted have created some confusion among pathologists and clinicians. Although a uniform terminology to classify neuroendocrine neoplasms arising in different systems has recently been proposed by WHO/IARC, some issues remain unsolved or need to be clarified. In this review, we discuss the lights and shadows of the current WHO classifications used to define and characterize NENs of the pituitary gland, lung, breast and those of the head and neck region, and digestive and urogenital systems.

## Introduction

Neuroendocrine neoplasms (NENs) are a heterogeneous group of epithelial neoplastic proliferations ranging from indolent well differentiated neuroendocrine tumors (NETs) to very aggressive poorly differentiated neuroendocrine carcinomas (NECs). They can arise virtually in any organ of the body and, although they show similar morphological and immunophenotypical features, they present some peculiar site-specific characteristics. The second decade of twenty-first century assisted to a terrific expansion of molecular technologies that has allowed an increasing insight into the pathogenetic mechanisms of NENs, as well as a greater understanding of their clinico-pathological relationships, and, last but not the least, the recognition of new prognostic (DAXX/ATRX, microsatellite instability, CD117 expression) and theranostic markers (somatostatin receptor subtype 2, deregulation in druggable pathways such as PI3K/AKT/mTOR and Notch signaling) [1]. In this context, several “hot topics” in the field of NENs classification have emerged. Synthetically, the most debated arguments have been: i) the conceptual separation between NETs and NECs, with the identification of distinct molecular pathogenetic pathways; ii) the existence of highly proliferating NETs, as well as their relationship and differential diagnosis with NECs; iii) the need to re-define the concept of mixed neuroendocrine/nonneuroendocrine neoplasms; iv) the growing exigence, from both the pathologists’ and the oncologists’ point of view, of a common framework for the nomenclature and classification of neoplasms arising in extra-GEP organs but showing overlapping morphological features with GEP NENs. These issues are better discussed, for each organ or system, in the following paragraphs. Here we present a general outline for each of the first four points, leaving the last one a specific discussion, later in the text.

Morphology represents the first cornerstone for the differential diagnosis between NET and NEC [2] and the combination of morphological features and Ki67 proliferative index improves the ability in this distinction, which has important clinical implications. Indeed, NETs and NECs should be considered as distinct clinico-pathological entities [2]. Molecular analysis has largely confirmed this assumption, showing that these two families of neoplasms recognize different pathogenetic pathways. In digestive NENs, the carcinogenesis of NECs seems to be strongly related to that of non-neuroendocrine carcinomas of the primary site in which they arise, with frequent inactivation of *TP53* and *RB1* [3–6]. In contrast, NETs of the GEP system exhibit unique molecular signatures, including, among other features, the inactivation of *MEN1*, *VHL*, *TSC1/2* genes, and the hyperactivation of the PI3K/mTOR pathway [6, 7]. In the lung, a similar situation has been demonstrated, but progression from NETs to NECs has been suggested in a subset of cases, with distinct clinico-pathological features [8, 9].

The existence of morphologically well differentiated NETs with a high proliferation index was not included either in the WHO classification of digestive NETs published in 2010 [10] or in the last WHO classification of lung tumors [11]. In fact, such tumors were classified as NECs based on the mitotic count and/or Ki67 proliferation index, according to the classification schemes. However, starting from clinical observations [12], it soon became evident that NENs with high proliferation index were morphologically, clinically and biologically heterogeneous, both in digestive and in thoracic sites [13–15] and the concept of NET G3 was integrated in the classification of GEP organs, leaving the definition of NEC to NENs with poorly differentiated morphology [16, 17].

Mixed neoplasms with neuroendocrine and non-neuroendocrine components, although rare, have been a matter of speculation under both diagnostic and therapeutic points of view. Indeed, their morphological heterogeneity underlies an intrinsic difficulty in making a correct diagnosis on small biopsy samples, as well as in not adequately sampled surgical specimens [18]. On the other hand, their protean biological nature must be taken into account when choosing the proper treatment. In order to better convey the diversity of this group of neoplasms that can be composed of different combinations of NENs (NET or NEC) and non-NENs (adenocarcinoma, acinar cell carcinoma, squamous cell carcinoma, and others) we proposed the term *Mixed neuroendocrine/non-neuroendocrine neoplasm (MiNEN)* [19]. The term MiNEN has been accepted in the WHO classification of GEP NENs [16, 17].

The existence of NENs in extra-thoracic and extra-digestive organs is a well-known, albeit rare, event. Head and neck and genitourinary tract are the commonest sites, but any epithelial organ may virtually be affected by a NEN. The diagnostic and therapeutic challenges of these NENs are related to their rarity, as well as to the heterogeneous classification schemes, which are not uniform in the various organs. To address this issue, and with the aim to settle down the bases of a robust and clinically significant nomenclature of NENs, a panel of expert pathologists, under the aegis of WHO and IARC, proposed, in 2018, a common classification frame for NENs [20]. This represents a milestone in the history of NENs, as it underlies the concept of a category of neoplasms that, with important site- and grade-related variations, retains substantial identity under morphological, biological and clinical points of view.

## Pituitary gland

**Fig. 1:**
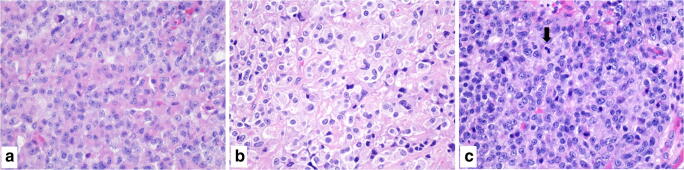
Morphology alone is not able to identify pituitary neuroendocrine tumors (PitNETs) that will behave in an indolent manner (**a**) or that will locally recur with signs of aggressiveness (**b**) or that will give metastatic dissemination (**c**), although in this latter case high cellularity and mitoses (arrow) are more frequently observed

Although the pituitary gland is small, it contains at least six neuroendocrine cells types secreting different hormones and bioactive peptides and this reflects the rather large number of different pituitary tumor types that can be found in the adenohypophysis. The pathological classification of anterior pituitary tumors has traditionally been bases on morphology, immunohistochemistry, and electron microscopy with the aim to correlate morphology and immunophenotype with function and clinical presentation [21]. With the advent of molecular techniques a large amount of new information has been obtained and the use of transcription factors involved in the lineage determination of different cell types has been proposed to classify anterior pituitary tumors [22]. This approach was the basis for the last WHO classification published in 2017 [16] that included two main tumor types: pituitary adenoma and pituitary carcinoma. Although this approach has the advantage to better characterize different pituitary tumor types based on cell lineage, it is not able to predict patients’ outcome. Indeed, this classification does not allow identifying with certitude those cases that will behave in an indolent manner and distinguishing them from those that will locally recur and need additional treatment, with a great impact on quality of life. In addition, this approach does not identify tumors that will give metastatic dissemination during follow-up, therefore deferring the diagnosis of pituitary carcinoma a posteriori*,* only when the presence of meningeal dissemination or metastatic spread will become clinically and/or radiologically evident (Fig. 1). Moreover, in the general attempt to conceptually unify NENs arising in the different anatomical sites, the need was felt to include also anterior pituitary tumors in the NENs family, to which they belong for morphological and functional reasons. Taken together, these considerations prompted a multidisciplinary group of experts in pituitary pathology to propose the new terminology “pituitary neuroendocrine tumor (PitNET)” instead of “pituitary adenoma” (Table 1) [23]. Thus, all anterior pituitary tumors are considered as lesions showing a potential clinical impact that can be additionally evaluated and better defined in terms of prognosis using a multiparametric approach including the Ki67 proliferative index and radiology appearance (Table 2) [24, 25]. Although this new approach appears appropriate and clinically useful since it reflects the real biology of these tumors, it is still matter of debate [26–28]. It is worth noting that the actual efficacy of this terminology has been supported by the WHO/IARC, which has recently proposed a common classification framework for neuroendocrine tumors to be used for all body sites [20].

**Table 1:** Specific pituitary neuroendocrine tumors (PitNETs) types*, transcription factor and hormone expression

**Cell type**	**TS**	**Hormone**	**Tumor type(s)**
**Corticotroph**	TPIT, NeuroD1	ACTH, β-endorphin, MSH	•DG corticotroph PitNET •SG corticotroph PitNET •Crooke cell PitNET
**Somatotroph**	PIT-1	GH	•DG somatotroph PitNET •SG somatotroph PitNET
**Mammosomatotroph**	PIT-1, ERα	GH, prolactin	•Mammosomatotroph PitNET •Mixed somatotroph/lactotroph PitNET
**Lactotroph**	PIT-1, ERα	Prolactin	•SG lactotroph PitNET •DG lactotroph PitNET •Acidophil stem cell PitNET
**Thyrotroph**	PIT-1, TEF, GATA2	TSH	•Thyrotroph PitNET
**Gonadotroph**	SF-1, ERα, GATA2	FSH, LH	•Gonadotroph PitNET
**Null cells**	None	None	•Null cell PitNET

*: in the current WHO classification [32], the term adenoma is used instead of PitNET; TS: transcription factor; DG: densely granulated; SG: sparsely granulated; ERα: estrogen receptor α

**Table 2:** Prognostic classification of pituitary neuroendocrine tumors Modified from Trouillas et al. 2013 [24]

Grade 1a: non-invasive PitNET	
Grade 1b: non-invasive and proliferative PitNET	
Grade 2a: invasive PitNET	
Grade 2b: invasive and proliferative PitNET	
Grade 3: metastatic PitNET (pituitary carcinoma)	

Invasion is defined as histological and/or radiological (MRI) signs of cavernous or sphenoid sinus invasion; Proliferation is considered on the presence of at least one of two criteria: Ki67 > 3% or mitoses >2/10HPF

## Head and neck

NENs of the head and neck are a group of heterogeneous epithelial neoplastic proliferations arising in virtually all the different organs of this region, including the nasal cavity, paranasal sinuses, nasopharynx, larynx, salivary glands, and middle ear. Their morphological and clinical features mainly depend on the degree of differentiation and on the site of origin and for these reasons they will be discussed separately in the following paragraphs.

In this region, the larynx is the commonest site of occurrence of NENs and both NETs and NECs (of small and large cell types) have been reported. The terminology used over the last years to define NENs of the larynx has been matter of debate [29]. In the WHO classification published in 2005, in analogy with NENs of the lung, they were subdivided into typical carcinoid, atypical carcinoid, and neuroendocrine carcinoma (small and large cell subtype) [30]. Unfortunately, the last edition of the WHO classification of tumors of the head and neck published in 2017 [31] changed this terminology resulting in a problematic and rather confusing scheme, as it misses several entities and is not in line with the terminology used for thoracic or digestive NENs. In this context, the most relevant issue regards the use of the term *neuroendocrine carcinoma* as a synonym for NEN, under the heading of which both morphologically well- poorly differentiated neoplasms are included [31]. This leads to a non-realistic framework, in which a three-tiered grading of so called “neuroendocrine carcinoma”, including well-differentiated, moderately differentiated and poorly differentiated neoplasms [31] introduces a continuum from very indolent to very aggressive neoplasms that is not supported by biological and genetic evidences [32, 33]. Consequently, the terminology used in the 2017 WHO classification to define neuroendocrine neoplasms clearly appears not appropriate and, most important for the clinical impact on the patient’s management, the use of the term neuroendocrine carcinoma to define a NET is dangerous because can be confounding for clinicians, who can be encouraged to use platinum-based chemotherapy to treat patients who would not benefit from it and would only experience severe collateral effects. Thus, we strongly recommend the use of the common classification framework for NENs also in this site, with NETs corresponding to typical and atypical carcinoids of the 2005 classification, and the term NEC reserved for morphologically poorly differentiated and clinically aggressive neoplasms [20, 29]. In addition, it is worth to be noted that in the 2017 WHO classification there is no mention on mixed neoplasms, which, on the contrary, have been described in the literature and may represent a diagnostic challenge for pathologists [19].

In the nasal cavity, the WHO classification only includes NECs [31], although the existence of NETs and mixed neuroendocrine-nonneuroendocrine neoplasms (MiNENs) has been well documented [19, 29, 34]. These two entities, although rare, need to be recognized because they show distinct prognosis and deserve a specific therapeutic approach.

A dedicated chapter on salivary gland NENs is not included in the 2017 WHO classification of head and neck tumors and they seem to be included in the chapter of poorly differentiated carcinoma, which also includes cases without a neuroendocrine differentiation [31]. Although the spectrum of the salivary gland NENs is almost totally covered by NECs of the small cell and large cell subtypes, a few cases of NETs have been reported and need to be considered among the possible differential diagnoses [29].

**Fig. 2:**
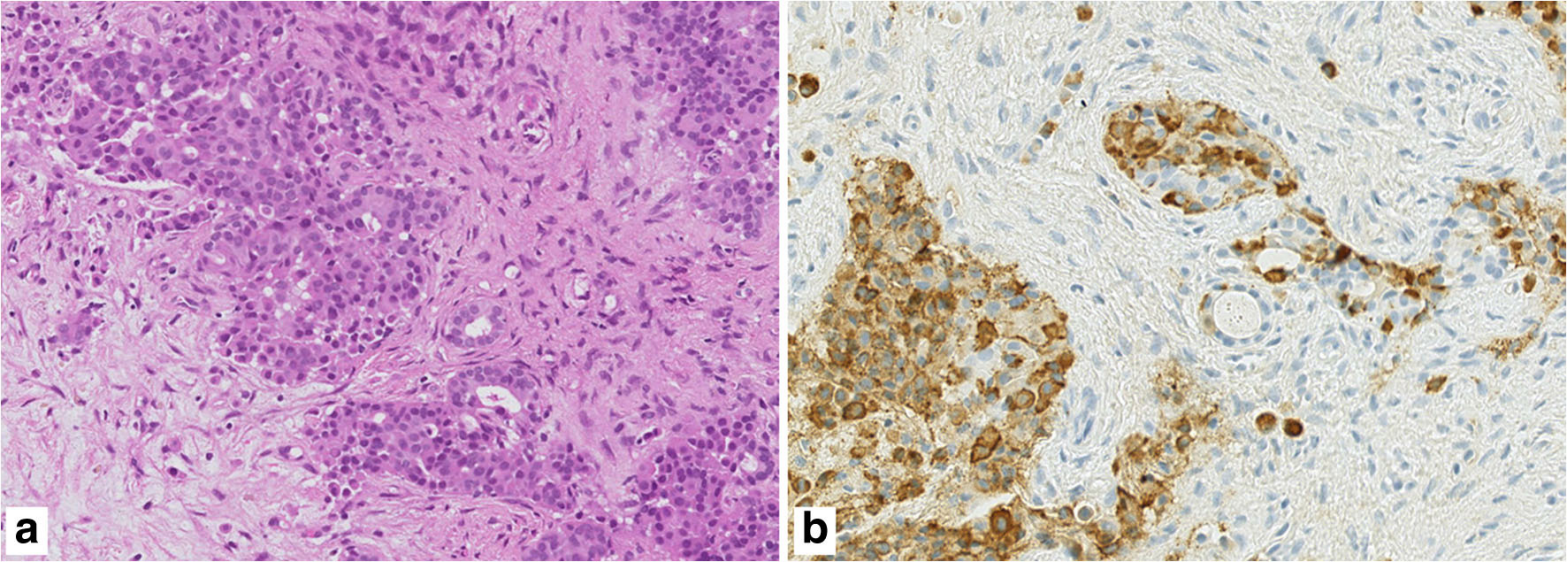
The so-called middle ear adenoma should be considered as a mixed neuroendocrine/nonneuroendocrine neoplasm, since it is composed of both a glandular (exocrine) and a solid (neuroendocrine) component (**a**), the latter positive for neuroendocrine markers including chromogranin (**b**)

The last entity to be considered among NENs of the head and neck is the so-called middle ear adenoma. Several studies have demonstrated that this tumor type is composed of both a glandular (exocrine) and solid (neuroendocrine) component, making the neuroendocrine tumor of the middle ear a mixed neoplasm (Fig. 2), for which the term MiNENs may be more appropriate [29].

In line with the recent classification framework supported by the WHO/IARC [20] the terminology used to define head and neck NEN needs to be revised and in Table 3 a classification scheme is proposed.

**Table 3:** Classification of epithelial head and neck neuroendocrine neoplasms

2005 WHO classification [30]	Current (2017) WHO classification [31]	Proposed new WHO classification [20]
Typical carcinoid	Well-differentiated neuroendocrine carcinoma, grade I	NET G1
Atypical carcinoid	Moderately differentiated neuroendocrine carcinoma, grade II	NET G2
Small-cell NEC and large cell NEC	Poorly differentiated neuroendocrine carcinoma, grade III	NEC (small and large cell types)
Combined small cell NEC	Neuroendocrine carcinoma with non small cell component (squamous cell carcinoma, adenocarcinoma, etc.)	MiNEN

## Lung

NENs of the lung are currently classified in four main categories, including typical carcinoid (TC), atypical carcinoid (AC), large cell neuroendocrine carcinoma (LCNEC) and small cell lung carcinoma (SmCLC) [11]. This classification is meant to be applied on surgical samples, and its mainstays are represented by morphological parameters: mitotic index, the presence of necrosis, and cell size, whereas Ki67 proliferation index is not included in the assessment for classification purposes. Although this terminology is not in line with the already mentioned common classification framework proposed by WHO/IARC [20], a substantial overlapping exists. In fact, TC and AC are considered well differentiated NENs (i.e., NETs), whereas LCNEC and SmCLC are regarded as poorly differentiated NENs (i.e., NECs). Besides these semantic issues, the practicing pathologist has experienced cases in which the clear-cut separation between, for example, TC and AC, or AC and LCNEC is not affordably allowed using the classical morphological parameters and additional workup is needed to reach a clinically meaningful diagnosis. In fact, Ki67 proliferation index has proven to be a useful parameter, at least in two different practical settings. First, on small biopsies with crash artifacts impairing the morphological evaluation, Ki67 may have paramount diagnostic value in distinguishing a NET (carcinoid) from a NEC (LCNEC or SmCLC) [35–39]. Second, Ki67 proliferation index has been shown to be a relevant prognostic factor in lung NETs (carcinoids) and its evaluation should be added to the pathological report, even if no agreement has been reached, until now, neither on the cut-off levels, nor on the possible integration with morphological parameters in a grading system similar to that of digestive NENs [40]. In addition, and importantly, NET (carcinoids) with high Ki67 proliferation index (between 10% and 20%) have been reported to have peculiar morphological and clinical features, that resemble those of digestive NET G3, and may represent a distinct type of aggressive well differentiated pulmonary NEN [41, 42].

The “molecular revolution” of the last decade has involved pulmonary NENs, as well. Based on the systematic review of compelling molecular evidences reported in literature, it has recently been proposed a molecular classification, which recognizes three different types of lung NENs, showing distinct molecular signatures and clinical behavior [43, 44]. In detail, they listed: 1) *primary high grade NENs*, which are the most frequent pulmonary NENs (70–75%), are diagnosed on small biopsies of heavy smokers, arise de novo with no recognizable precursor lesions, show classic SmCLC or LCNEC morphology, have low intra- and inter-tumor genetic heterogeneity with consistent inactivation of *TP53* and *RB1*, a high mutation burden, an extremely high Ki67 index, and a very aggressive clinical behavior, with no role for radical surgery; 2) *secondary high grade NENs*, which represent 20% to 25% of pulmonary NENs, arise in heavy smoker men, have variable morphology (AC, LCNEC, SmCLC), may show the presence of precursor lesions (neuroendocrine cell hyperplasia/DIPNECH, neuroepithelial bodies, carcinoids, non-small cell lung carcinoma), have high intra- and inter-tumor genetic heterogeneity with involvement of a variety of different pathways (inactivation of *TP53, RB1*, and *NOTCH*, *KRAS/LKB1/MEN1* mutation, *MYC, TERT, SDHA, RICTOR* amplification and epithelial-mesenchymal transition), suggesting a multistep pathogenesis, present a heterogeneous Ki67 index, and behave less aggressively than the previous type, being diagnosed mainly on surgical specimen after oncologically radical intervention; 3) *indolent low grade NENs*, which are the rarest type (5% of lung NENs), are diagnosed in non-smoker women, have well differentiated morphology (TC or AC), are often accompanied by precursor lesions (DIPNECH), may arise in MEN1 or other familial syndromes, show low mutation burden with involvement of chromatin remodeling genes, have an evenly low Ki67 index (10% or less), behave indolently and are successfully treated with surgery. In addition, a growing burden of evidence has been accumulating in support of the hypothesis that at least a subset of high grade NENs (NECs) in this site may arise from the progression of pre-existent NETs (carcinoids). In a recently published integrative analyses on 257 lung neuroendocrine neoplasms, it was possible to stratify atypical carcinoids into two prognostic groups with significantly different 10-year overall survival. Interestingly, a third group of neoplasms with carcinoid-like morphology but molecular profile closer to that of large cell NEC, defined supra-carcinoid, suggests a molecular link between carcinoids and large cell NECs also suggesting the possibility that a subset of large cell NEC may derive from pre-existing carcinoids [45].

The combination of morphological, proliferation and molecular parameters has leaded to the proposal of a comprehensive classification of lung NENs, which follows the common classification framework for NENs and has important clinical and therapeutic correlates [46].

## Breast

The issue of neuroendocrine differentiation in breast neoplasms has been a matter of debate since its first description in 1963 [47]. Indeed, although neuroendocrine phenotype has been demonstrated in a number of breast tumors using both immunohistochemical and ultrastructural methods [48–50], several morphological and genetic considerations prevent the full inclusion of so-called breast NENs in the common classification framework of NENs [51]. First, as the current classification of NENs is primarily based on morphological criteria that allow the distinction between NETs and NECs, a careful revision of the so-called breast NETs, as they are defined in the last WHO classification of breast tumors [52], shows that a definite and recognizable morphology is not identified, and the diagnosis, in practice, relies on immunostains for synaptophysin and chromogranin A, which are not enough to define a NEN [51]. Second, the genetic and expression profiles of so-called breast NETs is, in fact, overlapping with that of luminal A breast carcinomas and shares no similarities with those described for well differentiated NENs of other sites [53]. Third, the grading system for these neoplasms relies on the Elston-Ellis criteria and not on the proliferation rates as in NETs of other anatomical sites [52]. Fourth, which derives from the previous points, treatment strategies for the so-called breast NETs do not take in account the presence of neuroendocrine differentiation but follow standard protocols for the carcinomas of the breast of special and no special types [54]. Fifth, and last, there is no significant difference in the outcome of patients with so-called breast NETs, when compared to patients with non-neuroendocrine breast carcinomas of the same grade, stage and molecular profile [52]. Indeed, the therapeutic choices for so-called breast NET rely on predictive factors traditionally used for non-neuroendocrine breast cancer (hormone receptor expression, HER2 hyperexpression and amplification, and proliferative index) and targeted therapies for NETs of other sites (somatostatin analogues, mTOR inhibitors) have not proved to be effective on Br-NETs [55]. Indeed, although the expression of somatostatin receptors (SSRs) has been demonstrated in a subgroup of mammary NETs [56], several studies have demonstrated that SSRs, are also frequently expressed in breast carcinomas of luminal A type, removing any specificity of somatostatin analogues in the management of breast NET [57, 58].

A separate discussion is needed for breast NECs, which, albeit rare, represent a well-defined entity, showing morphological and clinical analogies with pulmonary and extra-pulmonary NECs. In these neoplasms, molecular studies have demonstrated the presence, in early pathogenetic steps, of alterations overlapping those of the non-endocrine breast carcinoma, similarly to what happens in other sites [59]. Indeed, these findings are paralleled by the morphological observation that, in many of these cases, the NEC component is associated with an in situ or invasive non-endocrine breast carcinoma [60]. For these reasons, breast NECs may be entitled to be included in the NENs category [51].

## Digestive system

**Fig. 3:**
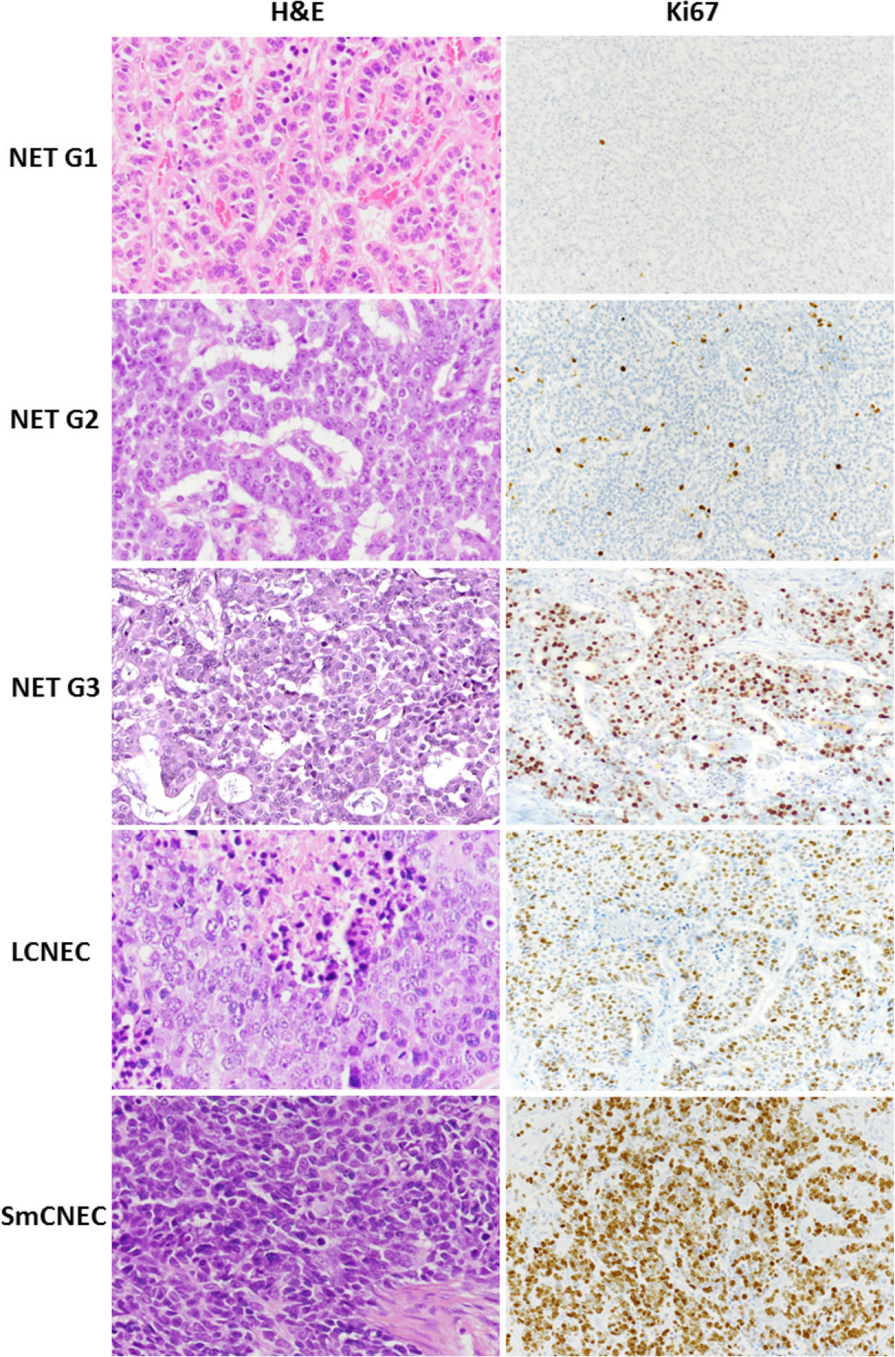
Morphology and Ki67 proliferation index of different NEN types of the digestive system. Neuroendocrine tumors (NETs) show a well differentiated morphology and on the basis of Ki67 labelling index they can be divided into G1, G2, and G3 category. Neuroendocrine carcinomas, both of large cell (LCNEC) and small cell (SmNEC) subtype, show poorly differentiated morphology and high Ki67 proliferative index. H&E: hematoxylin and eosin

The classification of digestive NENs has undergone a significant evolution over the last 20 years, reflecting the increasing knowledge on pathogenesis and molecular background of this heterogeneous group of neoplasms. The last WHO classification published in 2019 recapitulates these changes and integrates the most recent clinico-pathologic and molecular findings [17]. It is worth noting that, due to its easy application, good reproducibility, and great clinical significance, the classification approach for digestive NENs has been employed as the model for the common classification framework for NENs originating in different organs [20]. The basis of the classification includes the integration of both morphological (histological differentiation) and proliferative (grade) features and identifies three main groups (Table 4 and Fig. 3): well differentiated neuroendocrine tumor (NET), poorly differentiated neuroendocrine carcinoma (NEC), and mixed neuroendocrine/non-neuroendocrine neoplasm (MiNEN). The distinction between NET and NEC relies on morphology and includes specific cellular and architectural criteria [2]. NETs are then graded based on the proliferation index (mitotic count and Ki67-related proliferation index) and are divided in three groups (NET G1, NET G2, and NET G3), while NECs are by definition high grade neoplasms, and the specification G3 has been removed to avoid confusion with NET G3 (Table 4). This approach has proven to be of great help for the prognostic stratification of patients and it is particularly useful for the distinction, among the group of G3 neoplasms (Ki67 > 20%), between NET G3 and NEC, two entities showing distinct molecular background, clinical outcome, and therapeutic approach. This classification, based on the combination of both morphology and proliferation, appears as an important evolution of the WHO classification published in 2010, in which the distinction of NETs from NECs was mainly based on the proliferative index [10].

**Table 4:** WHO classification of digestive neuroendocrine neoplasms

	Morphological differentiation	Mitotic count/2mm^2^	Ki67 index
NET G1	well-differentiated	<2	<3%
NET G2	well-differentiated	2–20	3–20%
NET G3	well-differentiated	>20	>20%
NEC	poorly differentiated	>20	>20%
MiNENs	well or poorly differentiated	variable	variable

NET: neuroendocrine tumor; NEC: neuroendocrine carcinoma: MiNEN: mixed neuroendocrine/non-neuroendocrine neoplasm

In the current classification scheme, the identification of NEC is rather easy by combining morphology and proliferation, and its clinical and prognostic features seem to be similar, independently of the site of origin. Conversely, NETs show peculiar site-specific characteristics to be kept in mind and, for a better prognostic classification of tumors, the WHO classification needs to be integrated with other parameters, depending on the anatomical site. In this context, the prognostic role of Ki67 proliferative index deserves a discussion. Although the Ki67 proliferative index is a well-known prognostic marker, its prognostic role may vary depending on the tumor type and the site of origin [61].

In gastric NETs, the best prognostic stratification of patients is achieved by combining the clinico-pathologic subtype (type1, type 2, and type 3 gastric NETs), which is per se of prognostic value, with the Ki67 proliferative index, especially in patients with type 3 NETs [62].

In duodenal NETs, Ki67 has been demonstrated to be a predictor of lymph node metastasis and, although, associated with disease-specific survival at univariate analysis, it failed to be an independent prognostic factor discriminating survival between G1 and G2 tumors [63]. For this reason, a multiparametric approach including tumor size, site (ampulla versus other duodenal sites), and proliferative activity seems the most accurate to classify duodenal NETs.

Ileal NETs are peculiar tumors due to their ability to metastasize early to regional lymph nodes and/or the liver despite a low proliferation index (most tumors are G1). Consequently, it is conceivable that tumor grading fails to predict the metastatic potential of these tumors. However, Ki67 proliferative index is correlated with prognosis and, interestingly, it has been demonstrated that the increasing risk for tumor progression and tumor death for each increasing Ki67 unit was 14% and 18%, respectively [64]. This underlines the biological concept that Ki67 should be considered as a continuous variable and the calculation of the prognostic risk for each increasing Ki67 unit may be superior to the separation of tumors using fixed Ki67 categories, which, however, are useful to give a general classification approach.

Among digestive NETs, appendiceal ones are the most peculiar. Indeed, despite frequent infiltrative growth into the muscular layer and subserosa, lymph node metastases are rare and distant metastases are virtually absent [65]. Patients with these tumors, who often are young, have an excellent outcome after appendectomy. The Ki67 labeling index, which is generally low, has recently been demonstrated to predict nodal metastases together with size >1.5 cm and lympho-vascular invasion. However, the prognostic role of lymph node metastases is still matter of debate since no statistically different prognosis has been observed between patient with or without lymph node metastases [65–67]. For this reason, the survival benefit of right hemicolectomy is still unclear, and its choice needs to be demanded to multidisciplinary tumor boards in expert referral centers [66, 68].

About 90% of the rectal NETs are G1 which show better survival than G2 NETs [69], demonstrating the prognostic role of tumor grade and consequently of Ki67 proliferative index in these tumors. However, although tumor grade has been proved to be a prognostic marker in univariate analysis, it was not found as an independent factor at the multivariate analysis [69, 70]. For this reason, it has therefore been suggested that the best approach for stratifying patients into different prognostic categories seems to be multiparametric considering tumor grade together with tumor size, lympho-vascular invasion, level of wall infiltration, and immunophenotype (L-cell versus EC-cell NET) [69, 70], which have been demonstrated to have a prognostic role [71]. G1 rectal NETs with a size less than 10 mm, absence of lympho-vascular and muscular layer infiltration, and L-cell phenotype require only endoscopic resection, while larger G2 NETS, especially when of EC-cell type and deeply infiltrating the rectal wall in the presence of lympho-vascular invasion, need surgical resection.

Pancreatic NENs (PanNENs) are the tumors where the prognostic role of Ki67 proliferative index has been most investigated during the last years [72–75] and it represents an important prognostic marker together with stage [75]. The current WHO classification is very useful to stratify patients with PanNENs in different prognostic categories and its use is strongly recommended. It is worth noting that PanNETs less than 1 cm with a low proliferation index have been considered for a long time as benign tumors and, for this reason, the term pancreatic microadenoma has been proposed. However, as all other neuroendocrine tumors of the body, small and low proliferative PanNETs should be also considered malignant because they can give lymph node metastases [76].

In addition to NET and NEC, the WHO classification includes mixed neuroendocrine/ non-neuroendocrine neoplasms (MiNENs). The introduction of the term MiNEN, that we proposed for the first time in 2016 [19], represents an evolution in the definition of mixed neoplasms. Indeed, the term MANEC (mixed adenoneuroendocrine carcinoma) included in the previous WHO classification [10] did not convey the real spectrum of digestive mixed neoplasms creating confusion among pathologists and clinician [18]. The advantage of the term MiNEN resides in the fact that all different entities resulting from the different combinations of various neoplastic components can be included under this term, which represents an umbrella covering all different entities. Consequently, MiNEN should be regarded as a conceptual category, rather than a specific diagnosis. Indeed, in the pathology report, a diagnosis of MiNEN needs to be better specified including the correct identification and categorization of each component [18]. Despite this improvement, a main issue remains unsolved. By definition, the two components of a MiNEN should represent at least 30% of the tumor burden, but this cut-off was arbitrarily chosen presumably to assure that each component was quantitatively enough to influence the natural history of the disease and patient’s outcome. However, no systematic study has been performed, to date, to confirm the biological validity of this cut-off. The 30% cut-off seems a dangerous criterion, especially when a minor tumor component (<30%) is represented by a high grade NEC, which can drive patient’s prognosis independently on its percentage. Since in the last WHO classifications to define a MiNEN the two components are to be “morphologically recognizable” based on well-established criteria, the maintenance of the 30% cut off is probably not useful and/or essential. For this reason, further studies may help to solve this issue.

## Urogenital system

Neuroendocrine neoplasms (NENs) of the genitourinary tract are rare, but it is important to be aware of their existence as the correct diagnosis drives their treatment and prognosis. NENs have been described in the kidney, urinary bladder, prostate, testes, uterine cervix, uterine corpus, and ovaries. In all these organs, except for ovaries and testes, in which NETs are more frequent, the commonest NEN type is NEC. In the kidney and in the bladder, non-epithelial NENs (i.e. paragangliomas) have been described as well. The current classifications of tumors of the urinary tract and genital organs [77, 78] are partially in line with the common classification framework proposed by WHO/IARC [20]. Indeed, NENs of the kidney, of the urinary bladder and of the uterus are subdivided in NETs and NECs (with site-related variations), whereas in the prostate and in the gonads the nomenclature is still confusing and should be revised to adhere to the new criteria [77, 78]. In addition, the current classifications do not adequately recognize that most of poorly differentiated NENs of genitourinary tract are, in fact, combined with non-neuroendocrine components, and the category of MiNEN should be included in the classification scheme. Here, we will focus on bladder and prostatic NENs, which represent the main hot topic in the urogenital region, both because of their frequency and because of issue related to the nomenclature.

In the urinary bladder most NEN are small cell NECs, while the large cell subtype is exceedingly rare. Urinary bladder NECs are frequently associated with other carcinomatous components (urothelial and squamous carcinomas and adenocarcinoma), constituting bladder MiNENs. Since the poorly differentiated neuroendocrine component seems to drive the prognosis in MiNENs, its proper recognition is important for patients’ management. Immunohistochemical stains for synaptophysin and chromogranin A help to confirm the neuroendocrine differentiation of neoplastic cells, but additional markers can be used in discriminating and quantifying neuroendocrine versus non-neuroendocrine components. It has been demonstrated that NECs of the urinary bladder are consistently p16-positive, CK20-negative, GATA3-negative, and p63-negative, whereas high grade urothelial carcinomas show an opposite profile (p16-, p63+, GATA3+, and CK20+) [79, 80].

In the prostate, the presence of neuroendocrine differentiation, in the sense of general neuroendocrine markers expression, is relatively frequent and a significant subset of prostatic adenocarcinomas show immunoreactivity for synaptophysin, despite the absence of a true neuroendocrine morphology. Although these cases are listed by the last WHO classification as belonging to neuroendocrine neoplasms [77], they are not entitled to be part of the NENs category, as it is currently defined. Another entity that is included among prostatic neuroendocrine neoplasms is the so-called *adenocarcinoma with Paneth cell-like neuroendocrine differentiation*, which is defined as a prostatic adenocarcinoma with morphologically recognizable well-differentiated neuroendocrine cells showing cytoplasm stippled with brightly eosinophilic granules. The designation “Paneth cell-like” is a misnomer, as these granules do not contain lysozyme, as Paneth cells do, but are rather functionally and morphologically similar to neuroendocrine cells interspersed in the intestinal mucosa. Therefore, we prefer the designation of *adenocarcinoma with well differentiated neuroendocrine cells* for this entity and we consider that, when neuroendocrine cells are clustered in organoid structures that represent a significant proportion of the tumor mass, it should be regarded to as a MiNEN [81]. In this case, the presence of solid architecture in the neuroendocrine component should not be graded as Gleason pattern 5 [82]. Real NENs of the prostate are mainly represented by NECs. Prostatic NECs are uncommon neoplasms that may present in pure neuroendocrine form or as MiNENs, in association with prostatic adenocarcinoma. About half of cases occur in patients with a previous diagnosis of prostatic adenocarcinoma treated with androgen deprivation therapy, which has become castration-resistant. However, prostatic NEC may also occur de novo [81]. NETs are exceedingly rare in the prostate, and their existence itself is questioned. They may be diagnosed only when all the following criteria are satisfied: 1- presence of well differentiated neuroendocrine morphology; 2- absence of adenocarcinomatous component; 3- immunohistochemical expression of general neuroendocrine markers; 4- negativity of immunostainings for AR and PSA; 5- exclusion of prostatic metastasis or infiltration from a primary NET of another site. The main diagnostic problem on small biopsies is to distinguish prostatic NET from NEC, which has completely different prognostic implications [81]. The spectrum of prostatic neoplasms with neuroendocrine differentiation has been recently expanded by an entity called *prostatic carcinoma with amphicrine features*, an aggressive variant of prostatic carcinoma in which the totality of neoplastic cells present both a neuroendocrine and an exocrine phenotype [83]. However, it is important to recall that amphicrine neoplasms do not belong to the category of NENs, as they are currently defined [18].

## NENs of unknown primary origin

Virtually all NENs have metastatic potential and up to 20% of the cases present as metastasis from an occult primary [84–86]. The identification of the primary site is an important step towards the correct management of the patient, particularly when dealing with a NET since the therapeutic approach may vary depending on the site and cell type. In contrast, NECs, independent of the primary site, are currently treated with platinum-based regimens [87]. In this context, the role of the pathologist may be limited to the distinction between a visceral NEC and a Merkel cell carcinoma of the skin, because the latter requires wide local excision, sentinel node biopsy and, possibly, radiotherapy [88]. In contrast, thorough morphological and immunohistochemical analyses combined with imaging techniques are expected to give important clues to the recognition of the site of origin of a metastatic NET [89].

Among NETs, the tendency to metastasize is highest for those of pancreatic origin, followed by small intestinal, colonic, pulmonary and gastric neoplasms [87]. Irrespective of the primary site, the liver represents the most frequent location of metastatic NENs; lymph nodes, peritoneum, bone and lung represent further usual secondary sites [86]. However, virtually any body organ including those that can give rise to primary NENs may host metastatic NENs, including breast, ovary, thyroid, pancreas and pituitary [89]. Thus, it becomes evident that the diagnosis of a metastatic NEN gives rise to two orders of problems: (i) the identification of the occult primary site, and (ii) the distinction from a putative primary NEN of the organ in which the lesion is present. Both challenges are of crucial importance in the management of patients and the pathologist should be aware of the diagnostic tools to approach them and of the entities, which enter in the differential diagnosis. To address these issues, a coordinated and comprehensive clinical and pathological investigation is required. A diagnostic algorithm to identify the primary site of neuroendocrine neoplasms of unknown origin by using radiological and endoscopic methods along with radionuclear markers has been proposed [87]. From a pathological point of view, the critical employment of immunohistochemical stains for transcription factors and hormonal products has proved to be effective in identify the primary site of a metastatic NET, and the reader is referred to specific papers for detailed dissertation and diagnostic flowcharts [1]. Importantly, after the primary site has been identified, a careful grading and site-specific staging of the NET is crucial. In this context, the application of the common framework for the nomenclature and classification of NENs [20] shows, in our opinion its great value in guiding the physician hand in the management of these neoplasms, as the subdivision in NET, NEC and MiNEN families, independently of the primary site, provides an important initial characterization of the disease. Then, for the best treatment, this has to be obviously placed in the context of the specific site of origin of the NET.
